# IDENTIFYING ACUTE PRECIPITANTS AND CONSEQUENCES OF RACIAL/ETHNIC CONFLICTS IN RESIDENT-TO-RESIDENT AGGRESSION

**DOI:** 10.1093/geroni/igad104.0147

**Published:** 2023-12-21

**Authors:** E-Shien Chang, Jeanne Teresi, Mildred Ramirez, Joseph Eimicke, Sara Czaja, Karl Pillemer, Mark Lachs, Tony Rosen

**Affiliations:** Weill Cornell Medicine, New York City, New York, United States; Columbia University Stroud Center, New York City, New York, United States; Hebrew Home at RiverSpring Health, Riverdale, New York, United States; Research Division Of Hebrew Home At Riverdale, New York City, New York, United States; Weill Cornell Medicine, New York City, New York, United States; Cornell University, Ithaca, New York, United States; Weill Cornell Medicine, New York City, New York, United States; Weill Cornell Medicine, New York City, New York, United States

## Abstract

Resident-to-resident aggression (RRA) includes negative and aggressive physical, sexual, or verbal interactions between long-term care residents that in a community setting would likely be construed as unwelcome and have high potential to cause physical or psychological distress in the recipient. Though not explored in detail, previous research has shown that ethnic stereotyping may contribute to RRA. Our goal was to examine, for the first time, acute precipitants and consequences of RRA involving racial and ethnic conflicts. The parent study included comprehensive quantitative and qualitative information of 2,011 residents in 10 randomly-selected New York State nursing homes. Our qualitative analyses used an events perspective, focusing on how aggressive situations unfolded. We identified 51 single or repeated incidents of racial and ethnic conflicts during the study observation period. At the facility level, racial and ethnic conflicts were most commonly triggered by congested living spaces. At the relational level, racial and ethnic conflicts were frequently preceded by negative reactions to residents with cognitive impairment. At the individual level, racial and ethnic conflicts were found to be preceded by residents’ unmet or unrecognized needs. Unresolved racial and ethnic conflict commonly resulted in aggression escalation and spillover. Our results indicated racial and ethnic conflicts occurred commonly and repeatedly. Such behavior appeared tolerated and normalized, with limited institutional response. As the long-term care population becomes increasingly diverse, recognizing and addressing potential racial and ethnic conflicts becomes more pressing. Targeting racial and ethnic conflicts in RRA for intervention may also improve interactions between residents and frontline workers.

